# Washington University of Baltimore, Medical Department

**Published:** 1851-10

**Authors:** 


*Washington University of Baltimore, Medical Department.*—We have received the annual circular of this institution for 1851–2, and are pleased to find that the prospects of the faculty are more than ever cheering.

The gentlemen connected with this school are well known for high professional character and talent, and deserve the sympathy and support of the community. Competing with institutions largely endowed by the public money, they have, by personal efforts and private means, established themselves in a most favorable position. Their building is as elegant and commodious as could be desired, their ability undoubted by any, and their industry has been sufficiently proved by the obstacles they have already overcome.

We observe that the chair of surgery is filled by Dr. Valentine Mott, Jr., a gentleman of great surgical experience and skill, whose romantic patriotism as exhibited in the Sicilian resolution, has already made him known to the community.

We sincerely wish success to the Washington University of Baltimore.

